# Identification of HLA-A*2402-restricted HCMV immediate early-1 (IE-1) epitopes as targets for CD8+ HCMV-specific cytotoxic T lymphocytes

**DOI:** 10.1186/1479-5876-7-72

**Published:** 2009-08-23

**Authors:** Jong-Baeck Lim, Hyun Ok Kim, Seok Hoon Jeong, Joo Eun Ha, Sunphil Jang, Sang-Guk Lee, Kyungwon Lee, David Stroncek

**Affiliations:** 1 Department of Laboratory Medicine, Yonsei University College of Medicine, Seoul, South Korea; 2 Department of Transfusion Medicine, Warren G. Magnuson Clinical Center, National Institutes of Health, Bethesda, MD, USA

## Abstract

**Background:**

To identify novel HLA-A*2402-restricted human cytomegalovirus (HCMV) immediate early-1 (IE-1) epitopes for adoptive immunotherapy, we explored 120 overlapping 15-amino acid spanning IE-1.

**Methods:**

These peptides were screened by measuring the frequency of polyclonal CD8+ T cells producing intracellular interferon-γ (IFN-γ) using flow cytometry and the epitopes were validated with a HCMV-infected target Cr release cytotoxicity assay.

**Results:**

Initial screening was performed with 12 mini-pools of 10 consecutive peptides made from 120 overlapping peptides15-amino acids in length that spanned IE-1. When peripheral blood mononuclear cells (PBMCs) from HLA-A*2402 HCMV-seropositive donors were sensitized with each of the 12 mini-pools, mini-pools 1 and 2 induced the highest frequency of CD8+ cytotoxic T lymphocytes (CTLs) producing IFN-γ. When PBMCs were stimulated with each of the twenty peptides belonging to mini-pools 1 and 2, peptides IE-1_1–15_MESSAKRKMDPDNPD and IE-1_5–19_AKRKMDPDNPDEGPS induced the greatest quantities of IFN-γ production and cytotoxicity of HLA-matched HCMV-infected fibroblasts. To determine the exact HLA-A*2402-restricted epitopes within the two IE-1 proteins, we synthesized a total of twenty-one overlapping 9- or 10 amino acid peptides spanning IE-1_1–15 _and IE-1_5–19_. Peptide IE-1_3–12_SSAKRKMDPD induced the greatest quantities of IFN-γ production and target cell killing by CD8+ CTLs.

**Conclusion:**

HCMV IE-1_3–12_SSAKRKMDPD is a HLA-A*2402-restricted HCMV IE-1 epitope that can serve as a common target for CD8+ HCMV-specific CTLs.

## Background

Human cytomegalovirus (HCMV) infections occurring after allogeneic hematopoietic stem cell transplantation (HSCT) are frequently associated with high morbidity and mortality despite treatment with appropriate antiviral agents [1-3]. Cytotoxic T lymphocyte (CTL) responses have been known to correlate with recovery from HCMV disease in bone marrow transplant (BMT) recipients [4] and CD8+ CTLs are believed to play an important role in suppressing HCMV disease [5-7]. This has led to the development of clinical protocols whereby HCMV-specific CD8+ T cell clones are cultured from the transplant donor [8] and are administered to the transplant recipient. The adoptive transfer of these HCMV-specific CD8+ CTLs has proven to be effective in the prevention of reactivation and in the treatment of HCMV infections that are unresponsive to antiviral therapy [9-11].

Although HCMV protein pp65 is known to be an important target for HCMV-specific CTLs and 70% to 90% of all HCMV-specific CTLs recognize pp65 epitopes [12-14], CTLs specific for another HCMV protein, immediate early-1 (IE-1), occur in infected individuals at frequencies at least comparable to those of pp65-specific CD8+ T cells [15,16]. In addition, some recent studies have shown that the dominance and magnitude of the IE-1 specific CD8+ T cell response more strongly correlates with protection from HCMV disease than that of CD8+ T cell responses to pp65 [17-19].

Several alternative approaches have been used to generate antigen specific cytotoxic T cells. Antigen presenting cells (APCs) have been genetically modified to which express HCMV pp65 [20,21]. Epstein-Barr virus (EBV)-transformed B lymphoblastic cell lines (EBV-BLCL) have been used to generate EBV-specific CTLs. Genetic manipulation of APCs including dendritic cells (DCs) as well as EBV-BLCL result in the natural processing and presentation of HCMV and EBV antigens but their clinical use is complicated by regulatory issues, high cost, and the long duration of time required to qualify viral supernatants and cell therapy products [22].

Several reports have proposed the use of donor-derived HCMV-specific T cells generated by sensitization with HCMV lysates loaded on either donor peripheral blood mononuclear cells (PBMCs) or monocyte-derived cytokine activated dendritic cells [7,8]. However, concerns have been raised by regulatory agencies regarding the possibility that lysates of HCMV-infected cells might contain live viral particles that could be transferred to the host and HCMV T cells expanded using viral lysate may be predominantly CD4+ cells [7].

The use of immune-dominant HCMV peptides is another alternative for adoptive immune therapy. Adoptive immune therapy with peptides is feasible as demonstrated by the use of several HCMV-specific peptides derived from pp65 protein to expand large quantities of HCMV-specific CTLs [9,23].

The immune dominance of pp65 and IE-1 proteins among HCMV antigens has been reported, but the number of previously identified CTL epitopes derived from IE-1 protein is limited. The wide clinical application of HCMV-peptide, HLA-restricted, adoptive immune therapy requires the identification of at least one immune dominant HCMV pp65 and IE-1 peptide for each class I HLA antigen. Especially for epitopes such as HLA-A*2402 which is the most frequent HLA-A allele in many different races. To this aim, we report a new HLA-A*2402-restricted pentadecamer peptide from HCMV IE-1, IE-1_3–12_SSAKRKMDPD, that can be used to stimulate cytotoxic T cells for adoptive immunotherapy.

## Methods

### Donor collection and cell preparation

Peripheral blood mononuclear cells (PBMCs) were collected from nineteen HLA-A*2402 donors who were HCMV seropositive. The presence of IgG and IgM HCMV antibodies in each donor was analyzed by passive latex agglutination (CMVSCAN kit, Becton-Dickinson Microbiology System, Cockeysville, MD). MHC Class I genotypes were determined by sequence-specific primer PCR using genomic DNA by the HLA laboratory at the Seoul Clinical Laboratory (Seoul, Korea). PBMCs were isolated from the donors' peripheral blood by density-gradient centrifugation using Ficoll-Hypaque 1.077 (Pharmacia Biotech, Wilkstrom, Sweden). The mononuclear cells were washed twice with phosphate buffered saline (PBS, Gibco, Grand Island, NY) and cryopreserved at -160°C in human AB+ serum and basal Iscove's medium (Gibco, Grand Island, NY) containing 10% DMSO (Sigma, St. Louis, MO). This research was approved by the institutional review board of Yonsei University Health System and all participants gave written informed consent.

### Peptide libraries and study design

**Figure 1:**
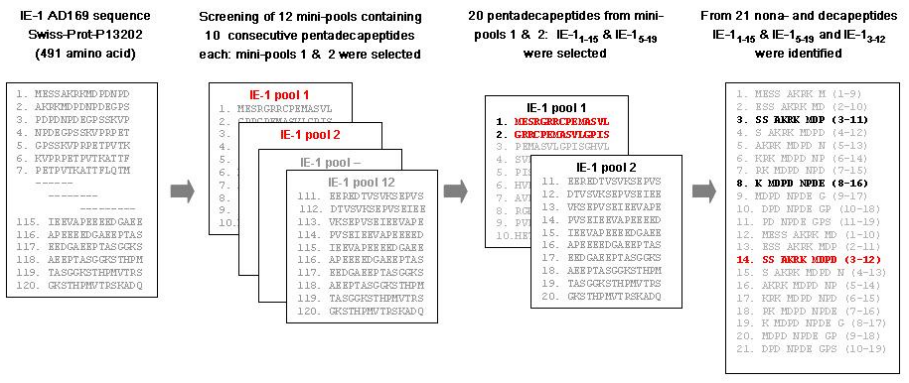
**IE-1 peptide library and study design**. The library of peptides spanning HCMV IE-1 was made up of 120 peptides 15 amino acids in length that overlapped by 11 residues which were used to make 12 mini-pools each containing 10 consecutive peptides. We screened and choose the most immunogenic mini-pools by quantifying IFN-γ production by stimulated CD8+ CTLs using intracellular flow cytometry analysis. After finding that mini-pools 1 and 2 were the most potent stimulators of IFN-γ, we screened and choose the best 15-amino acid peptides among twenty 15-amino acid peptides belonging to these two mini-pools by quantifying IFN-γ production by peptide stimulated CD8+ CTLs using flow cytometry and a HCMV-infected target cell killing assay. Next, we identified the exact HLA class I restricted-HLA-A*2402 epitope by screening a total of twenty-one overlapping nona- or decamer peptides spanning selected 15-amino acid peptides. These 21 peptides were also tested by intracellular flow cytometry and cytotoxicity assays.

Peptide library for HCMV IE-1 protein were made up of 15-amino acids in length that overlapped by 11 residues and covered the complete IE-1 protein (CMV AD169) [24]. The entire IE-1 library was made up of 120 peptides and these were commercially synthesized (A & Pep, Yoengi-gun, Korea). The peptides were diluted in DMSO to working solution concentrations and pooled into mini-pools containing 10 consecutive peptides each. For IE-1, 12 mini-pools of 10 peptides were made. We screened and choose the most immunogenic mini-pools among the 12 mini-pools by quantifying the IFN-γ production from the stimulated CD8+ CTLs using flow cytometry as described below. We then screened and identified the best 15-amino acid peptides among the twenty 15-amino acid peptides belonging to the selected mini-pools by quantifying the IFN-γ production from the stimulated CD8+ CTLs using flow cytometry and HCMV-infected target cell killing assay as described below. For further identification of the exact HLA class I restricted-HLA-A*2402 epitopes, we tested a total of twenty-one overlapping nona- or decamer peptides spanning selected 15-amino acid peptides by quantifying the IFN-γ production from the stimulated CD8+ CTLs using flow cytometry and HCMV-infected target cell killing assay again. Figure 1 briefly shows the study design.

### Generation of autologous dendritic cells (DCs) from PBMCs

Peptide-loaded autologous DCs were generated as previously described [3,25]. PBMCs obtained after Ficoll-Hypaque centrifugation were incubated for 2 hours at 37°C in complete RPMI medium. Adherent monocytes were resuspended at a concentration of 5 × 10^6^/mL in serum-free medium, supplemented with GM-CSF (1500 IU/mL, Pepro Tech Inc., Rocky Hill, NJ) and IL-4 (1200 IU/mL, Pepro Tech Inc., Rocky Hill, NJ). On days 2, 4, and 6 of culture, fresh cytokines were added. Fresh medium was added depending on cell growth. On day 7 of culture, 10 ng/mL tumor necrosis necrosis factor-α (TNF-α, R&D Systems, Minneapolis, MN) was added for the maturation of the DCs. After 72-hour maturation, autologous DCs were pulsed with peptides for at least 3 hours.

### Generation of peptide-specific polyclonal CTLs

PBMCs from HCMV-seropositive donors were plated at a concentration of 2 × 10^6 ^cells per well in a 24-well culture plate (Nunc, Roskilde, Denmark) with 2 mL of medium and directly stimulated with peptides at a concentration of 10 μg/mL/well (on day 1) and with peptide-pulsed autologous DCs (4~10 × 10^6^/well, on day 7 for a 1- or 2-week expansion for flow cytometry analysis or cytotoxicity assay, respectively). Recombinant human interleukin-2 (rhIL-2, 100 IU/mL, Pepro Tech Inc., Rocky Hill, NJ) was added to the culture every other day and the cells were cultured for 14 days.

### Detection of IFN-γ producing CD8+ T cells in response to peptide stimulation by flow cytometry

Two- week peptide-expanded PBMCs (1 × 10^6^) stimulated with PHA (Sigma, St. Louis, MO) and PBMCs stimulated with autologous DCs that were not loaded with any peptide were used as positive and negative controls respectively. HCMV pp65_495–503 _(NLVPMVATV, HLA-A*0201) or pp65_341–350 _(QYDPVAALFF, HLA-A*2402), pp65_91–100 _(SVNVHNPTGR, HLA-A33) were used as positive or negative controls according to donor's HLA type [3,24]. One hour after stimulation, 10 mg of Brefeldin A (Sigma, St. Louis, MO) was added to each well. After 5 additional hours of incubation, PBMCs were washed once with PBS and were then incubated in PBS containing 1 mM EDTA for 10 minutes. After two further washes with PBS and 5% fetal calf serum (FCS, Biosource International, Rockville, MD) the cells were incubated with fluorescence-labeled monoclonal antibodies for 15 minutes on ice in the dark. Staining and analysis was performed as previously described [3,14,26].

### Antibodies and flow cytometry analysis

FITC-conjugated anti-IFN-γ, PerCP-conjugated anti-CD69, PerCP-conjugated anti-CD3 and PE-conjugated anti-CD8 were purchased from BD Biosciences. Per sample, 50,000–100,000 events in the FSC/SSC lymphocyte gate were acquired on a FACS Calibur flow cytometer (Becton Dickinson, San Jose, CA). For data analysis (CELLQuest software; Becton Dickinson), CD3+/CD8+ events were displayed in a CD69+ versus IFN-γ dot plot. CD8+/IFN-γ cells were expressed as a percent of the respective reference population. The assessment of responses was previously described in more detail [3,14,26].

### Fibroblast cell lines as target cells

Fibroblasts from allogeneic donor (HLA-A*2402) derived skin biopsies were used as target cells. The fibroblasts were propagated in MEM-α supplemented with 1% NEAA (nonessential amino acid, Sigma, St. Louis, MO), 10% fetal calf serum, and antibiotics. AD-169 HCMV strain (VR-538, American Type Culture Collection, Manassas, VA) was propagated in fibroblasts and the infected cultures were harvested when a cytopathic effect was evident. The cells were spun at 1500 rpm for 10 minutes and aliquots of supernatant were stored at -80°C until use. HCMV infectivity of the fibroblasts was confirmed by HCMV-specific real time RT-PCR testing that targeted the HCMV IE-1 antigen (Roche, Nutley, NJ)

### Cytotoxicity assays

Cytotoxicity assays were performed employing ^51^Cr release as previously described [27,28]. Briefly, HCMV-infected fibroblasts were labeled overnight with ^51^Cr (100 mCi/10^6 ^cells; PerkinElmer Life and Analytical Science, Waltharn, MA), washed in PBS, and dispensed in triplicate into 96-well V-bottom plates (Nunc, Roskilde, Denmark) at 4 × 10^3 ^cells/well. CTLs were added to the infected fibroblasts at an effector to target cell ratio of 10:1, 30:1, 50:1 and 100:1. The cells were pelleted and after a 5 hour incubation period the supernatant was analyzed in a gamma counter. Spontaneous and total release counts for each well were used to calculate percent specific release with the following formula: % specific release = (experimental cpm - spontaneous cpm)/(total cpm - spontaneous cpm).

## Results

### Screening IE-1 peptide mini-pools by induction of IFN-γ production by CD8+T cells

**Figure 2:**
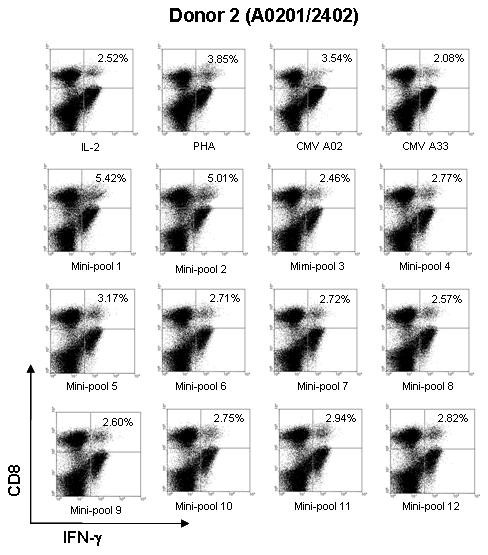
**Results of screening of the 12 peptide mini-pools by quantifying intracellular IFN-γ by CD8+T cells**. To select the most potential immune-dominant epitopes PBMCs from five HLA-A*2402 HCMV-seropositive donors (Donors 1–5) were stimulated with each of the 12 mini-pools and intracellular IFN-γ production was measured by flow cytometry. The results of testing cells from Donor 2 who expressed HLA-A*0201/2402 are shown. Peptide mini-pools 1 and 2 showed a higher frequency of IFN-γ accumulation by CD8+ T cells than the other mini-pools. Therefore, mini-pools 1 and 2 were selected for further study. PHA and HCMV A2 (pp65_495–503_) peptide-stimulated PBMCs were used as positive controls and HCMV A33 (pp65_91–100_) peptide and IL-2 only stimulated PBMCs (IL-2) were used as negative controls.

To determine which of the 12 mini-pools contained potential immune dominant candidate peptides, PBMCs from five HLA-A*2402 HCMV-seropositive donors (donor 1–5) were stimulated with each of the 12 mini-pools. Intracellular IFN-γ production was measured by flow cytometry. As a positive control, PBMCs from HCMV-seropositive donors were stimulated with both phytohemaglutinin (PHA) and pp65_328–335 _(QYDPVAALF, HLA-A*2402) [24]. In addition, PBMCs from donors incubated without any peptide or with pp65_91–100 _(SVNVHNPTGR, HLA-A33) [3] were used as negative controls. Among the 12 mini-pools, mini-pool 1 induced a greater frequency of IFN-γ producing CD8+ cytotoxic T cells than mini-pools 3 through 12 in four of the five donors. In addition, mini-pool 2 induced a higher frequency of IFN-γ producing CD8+ cytotoxic T cells than mini-pools 3 through 12 in three of the five donors. Therefore, both peptide mini-pools 1 and 2 were selected for further study. A representative experiment is illustrated in Figure 2.

### Identification of specific 15-amino acid candidate eptitopes by in vitro sensitization and induction of IFN-γ production

**Figure 3:**
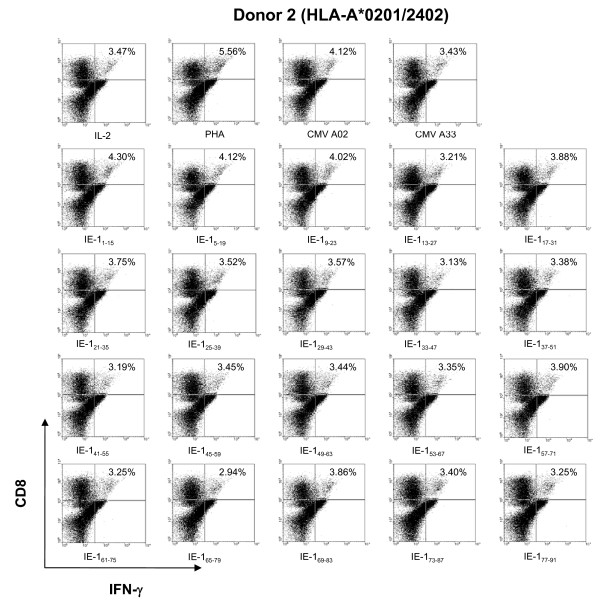
Intracellular IFN-γ protein production by HLA-A*2402 CD8+ CTLs stimulated with the twenty individual 15-amino acid peptides included in mini-pools 1 and 2. To determine which 15-amino acid peptides belonging to mini-pools 1 and 2 had the capacity to specifically re-induce CTL immune activity, intracellular IFN-γ production by CD8+ T cells was measured in HCMV-seropositive HLA-A*2402 cells from five donors (Donors 2, 3, and 6–8) that had been *in vitro *sensitized for a week with each of the twenty candidate 15-amino acid peptides. The results of testing cells from Donor 2 are shown. Peptides IE-1_1–15_MESSAKRKMDPDNPD and IE-1_5–19_AKRKMDPDNPDEGPS consistently induced greater quantities of IFN-γ protein production than the other 15-amino acid peptides tested. PHA and HCMV A2 (pp65_495–503_) peptide-stimulated PBMCs were used as positive control and HCMV A33 (pp65_91–100_) peptide and IL-2 only stimulated PBMCs (IL-2) were used as negative controls.

To determine which 15-amino acid peptides belonging to mini-pools 1 and 2 had the capacity to specifically re-induce CTL immune activity, intracellular IFN-γ production of CD8+ T cells was measured in HCMV-seropositive HLA-A*2402 cells from five donors (Donors 2, 3, and 6–8) that had been *in vitro *sensitized for a week with each of the twenty candidate 15-amino acid peptides. After a one week *in vitro *sensitization PBMCs were restimulated with dendritic cells derived from autologous monocytes which were loaded with each of the twenty 15-amino acid peptides. After a 6-hour resensitization, intracellular IFN-γ protein production by CD8+ T cells from the HCMV-seropositve HLA-A*2402 donors was measured by intracellular flow cytometry. In a representative experiment illustrated in Figure 3, in all donors peptides IE-1_1–15_MESSAKRKMDPDNPD and IE-1_5–19_AKRKMDPDNPDEGPS consistently induced greater quantities of IFN-γ production than the other 15-amino acid peptides tested. As a control, the PBMCs were also sensitized *in vitro *for a week with the HLA-A*2402-restricted epitope, pp65_328–335_QYDPVAALF and the HLA-A*0201-restricted epitope, pp65_495–503_NLVPMVATV [14] as positive controls and with the HLA-A*3303-restricted epitope, pp65_91–100 _SVNVHNPTGR, as a negative control.

These results suggest that IE-1_1–15_MESSAKRKMDPDNPD and IE-1_5–19_AKRKMDPDNPDEGPS are potential HLA-A*2402-restricted HCMV IE-1 epitopes and both peptides were selected for further study.

### Analysis of the peptide-specific cytotoxicity of the two 15 amino acid peptides

**Figure 4:**
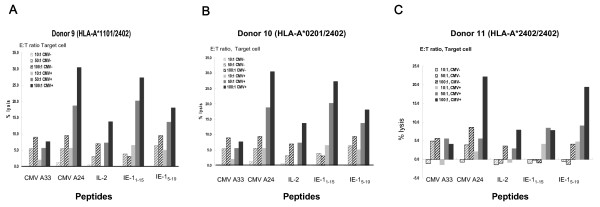
**Cytotoxic effects of IE-1_1–15 _and IE-1_5–19 _peptide-specific CTLs against CMV-infected fibroblast**. PBMCs from three HLA-A*2402 HCMV-seropositive donors (Donors 9,10 and11) were sensitized *in vitro *for two weeks with IE-1_1–15_MESSAKRKMDPDNPD and IE-1_5–19 _AKRKMDPDNPDEGPS and the *in vitro *sensitized cells were tested for cytotoxicity against HLA-matched HCMV-infected fibroblast. The cytotoxicity assay was carried out by measuring ^51^Cr release from HLA-A*2402 HCMV-infected fibroblasts. PBMCs from Donor 9 (Figure 4A) and Donor 10 (Figure 4B) that were *in vitro *sensitized for 2 weeks with IE-1_1–15 _MESSAKRKMDPDNPD were highly cytotoxic to HLA-A*2402 HCMV-infected fibroblasts causing as much targeted cell lysis as PBMCs sensitized with a positive control. However, in Donor 11, IE-1_5–19_AKRKMDPDNPDEGPS showed higher cytotoxicity to HCMV-infected fibroblasts than that of IE-1_1–15_MESSAKRKMDPDNPD (Figure 4C). PMBCs stimulated with the HLA-A24-restricted HCMV pp65 epitope HCMV A24 (pp65_341–350_) was used as positive control and PBMCs stimulated with the HCMV-A33 restricted epitope CMV A33 (pp65_91–100_) peptide and PBMCs simulated only with IL-2 (IL-2) were used as negative controls.

To confirm that IE-1_1–15_MESSAKRKMDPDNPD and IE-1_5–19_AKRKMDPDNPDEGPS are immune dominated peptides for HLA-A*2402 subjects, PBMCs from three HLA-A*2402 HCMV-seropositive donors (Donors 9, 10 and 11) were sensitized *in vitro *for two weeks with the candidate pentadecapeptides. The *in vitro *sensitized cells were tested for cytotoxicity against HLA-matched HCMV-infected targets. The cytotoxicity assay was carried out by measuring ^51^Cr release from HLA-A*2402 HCMV-infected fibroblasts. For all three donors tested IE-1_1–15_MESSAKRKMDPDNPD- and IE-1_5–19_AKRKMDPDNPDEGPS-sensitized CTLs lysed greater quantities of HCMV-infected fibroblasts than the negative control cells. PBMCs from donors 9 and 10 that were *in vitro *sensitized for 2 weeks with IE-1_1–15_MESSAKRKMDPDNPD were highly cytotoxic to HLA-A*2402 HCMV-infected fibroblasts. PBMCs *in vitro *sensitized with IE-1_1–15_MESSAKRKMDPDNPD lyzed a similar proportion of HCMV-infected fibroblasts as PBMCs sensitized with pp65_495–503 _which was used as a positive control (Figure 4A, B). However, in donor 11 IE-1_5–19_AKRKMDPDNPDEGPS showed higher cytotoxicity to HLA-A*2402 HCMV-infected fibroblasts than that of IE-1_1–15_MESSAKRKMDPDNPD (Figure 4C). These results confirmed that both of IE-1_1–15_MESSAKRKMDPDNPD and IE-1_5–19_AKRKMDPDNPDEGPS were likely to be the best immunogenic epitopes for HLA-A*2402 among HCMV IE-1 proteins. Next, we identified the most immunogenic nona- or decarmer MHC class I-restricted peptides spanning IE-1_1–15 _and IE-1_5–19 _using a HCMV-infected fibroblast cytotoxicity assay.

### *Ex vivo *sensitization with 9- and 10 amino acid peptides spanning IE-_11–15 _and IE-_15–19_

**Figure 5:**
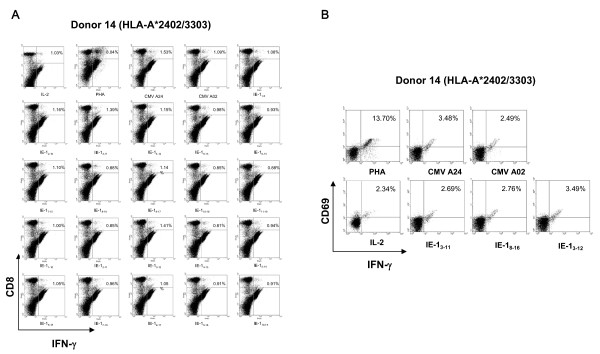
**Intracellular IFN-γ analysis of IE-1_1–15 _and IE-1_5–19 _derived HCMV-specific CTLs**. To determine the exact HLA class I restricted- HLA-A*2402 specific IE-1 epitopes, we synthesized a total of twenty-one overlapping nona- or decamer peptides spanning IE-1_1–15 _and IE-1_5–19_. Intracellular IFN-γ protein production was measured in six HCMV-seropositive HLA-A*2402 donors (Donors 12–17). Among the twenty-one candidate peptides, IE-1_3–11_SSAKRKMDP, IE-1_3–12_SSAKRKMDPD and IE-1_8–16_KMDPDNPDE peptide induced the highest quantities of IFN-γ protein production. Peptide IE-1_3–12_SSAKRKMDPD induced the greatest quantities of IFN-γ production in five of six donors. The results of testing Donor 14 are shown. The peptide IE-1_3–12_SSAKRKMDPD induced higher quantities of IFN-γ production by CD8+ CTLs from Donor 14 than any of the other peptides tested (Panel A). In addition, this peptide also induced the greatest quantities of IFN-γ protein production by CD8+CD69+ CTLs (Panel B). PHA and CMV A24 (pp65_341–350_) peptide-stimulated PBMCs were used as positive control and CMV A2 (pp65_495–503_) peptide and IL-2 only stimulated PBMCs (IL-2) were used as negative controls.

To determine the exact HLA class I restricted HCMV IE-1 protein epitopes that were immunogenic in HLA-A*2402 subjects, we synthesized and tested a total of twenty-one overlapping nona- or decamer peptides spanning IE-1_1–15 _and IE-1_5–19_. Intracellular IFN-γ protein production was measured in cells from seven HCMV-seropositive HLA-A*2402 donors (Donors 12–18) that had been *in vitro *sensitized for 2 weeks with each of the twenty-one candidate peptides. Among the twenty-one candidate peptides, IE-1_3–11_SSAKRKMDP, IE-1_3–12_SSAKRKMDPD and IE-1_8–16_KMDPDNPDE induced greater quantities of IFN-γ production than the other peptides tested. Peptide IE-1_3–12_SSAKRKMDPD was especially potent. It induced greater quantities of IFN-γ production than the other two peptides in six of seven donors. Therefore, IE-1_3–12_SSAKRKMDPD was likely the most immunogenic HLA-A*2402 epitope within HCMV IE-1. A representative experiment using cells from donor 14 is illustrated in Figures 5A and 5B. The response of donor 14's CD8+ cells to IE-1_8–16_KMDPDNPDE was weak (Figure 5A), but IE-1_8–16_KMDPDNPDE stimulated significant quantities of IFN-γ in CD8+ cells from five of the seven HLA-A*2402 expressing donors tested.

### HCMV IE-_13–12 _SSAKRKMDPD specific cytotoxicity

**Figure 6:**
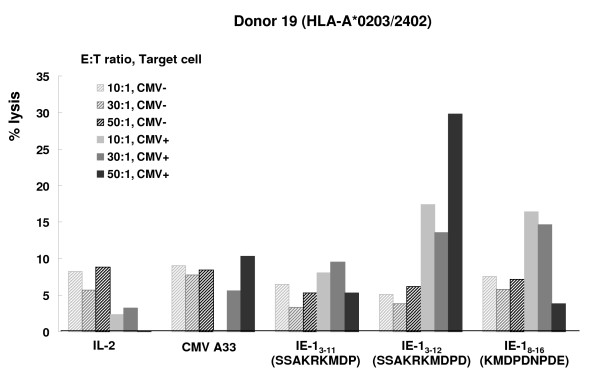
**HCMV IE-1_3–12 _SSAKRKMDPD specific cytotoxicity**. PBMCs from donors expressing HLA-A*2402 (Donor 19) were sensitized *in vitro *for 2 weeks with IE-1_3–11_SSAKRKMDP, IE-1_3–12_SSAKRKMDPD and IE-1_8–16 _KMDPDNPDE and tested for cytotoxicity using a ^51^Cr release assay against HLA-matched HCMV infected fibroblasts. The IE-1_3–12_SSAKRKMDPD sensitized CTLs lysed greater quantities of HCMV-infected fibroblasts than the negative controls. HCMV A33 (pp65_341–350_) peptide and IL-2 only stimulated PBMCs (IL-2) were used as negative controls.

To provide further evidence that IE-1_3–12_SSAKRKMDPD induced epitope-specific and HLA-A*2402-restricted cytotoxicity, PBMCs from a donor expressing HLA-A*2402 (Donor 19) were sensitized *in vitro *for 2 weeks with IE-1_3–11_SSAKRKMDP, IE-1_3–12 _SSAKRKMDPD and IE-1_8–16_KMDPDNPDE. The *in vitro *sensitized cells were tested for cytotoxicity using a ^51^Cr release assay against HLA-matched HCMV-infected targets. IE-1_3–12 _SSAKRKMDPD-sensitized CTLs lysed greater quantities of HCMV-infected fibroblasts than the negative control cells. CTLs sensitized with IE-1_5–16_KMDPDNPDE also lysed greater quantities of HCMV-infected fibroblasts than the negative control cells, but they lysed less HCMV-infected fibroblasts than CTLs sensitized with IE-1_3–12_SSAKRKMDPD (Figure 6).

## Discussion

This study focused on the identification of novel HLA-A*2402 CTL epitopes derived from HCMV IE-1 protein using pools of overlapping 15-amino acid peptides. These HCMV-specific HLA-restricted epitopes will be useful for vaccination, adoptive immunotherapy, and the monitoring of cellular immune response against HCMV disease in transplant recipients.

Over the last decade vaccination strategies using the immunogenic peptides derived from several HCMV proteins have been successful in preventing the reactivation of latent HCMV infection [17-19]. One of the most important steps in a peptide vaccine approach is the identification of immunogenic epitopes within HCMV proteins, which bind to HLA Class I molecules that are expressed by a major proportion of the population [29,30]. Although HLA-A24 is the most frequent HLA-A antigen among Asians, HLA-A24-restricted HCMV IE-1 epitopes have not yet been described.

Many current strategies for selecting potentially immunogenic epitopes are based on the use of algorithms that predict the binding affinities of specific peptides to HLA Class I molecules. Peptides predicted to have a high binding affinity are tested for their ability to sensitize CTLs. This strategy can be a very effective way of identifying new immune dominant peptides, but it has been useful for only a limited number of peptide sequences and HLA alleles [31,32]. Furthermore, as demonstrated by Elkington et al, even for those HCMV-pp65 peptides that were predicted to bind to common HLA alleles, only 40% elicited cytokine-producing T cells detected by enzyme linked immunospot (ELISPOT) assays, and only a subset of the T-cell lines generated from HLA-A*0201-seropositive donors in response to these peptides actually lysed HCMV-infected cells [33]. We have explored another method to identify HLA-A24-restricted HCMV IE-1 epitopes. Pools of overlapping 15-amino acid peptides spanning the sequence of HCMV IE-1 were used for sensitization and generation of HCMV-specific T cells. Such 15-amino acid peptides previously have been used to identify immunogenic viral epitopes recognized by T cells in the blood of healthy individuals and allograft recipients [25]. By analysis of responses to intersecting mini-pools, specific 15-amino acid peptides containing immunogenic epitopes were identified and the epitopes subsequently defined by testing responses to individual 9 or 10 amino acid sequences contained in these 15-amino acid peptides [26].

In our study a total of twelve mini-pools contained 10 consecutive 15-amino acid peptides were prepared using one hundred-twenty 15-amino acid peptides spanning HCMV IE-1 protein. The peptide pools were screened by quantifying the production of IFN-γ by CD8+ T cells from four HLA-A*2402 donors using flow cytotometry analysis. Mini-pool 1 (Donors 1, 2, 3, and 4) and mini-pool 2 (Donors 1 and 2) induced higher frequencies of CD8+ T cells producing IFN-γ than the other mini-pools. Mini-pools 5, 7 and 9 showed a higher frequency IFN-γ production in a single donor (Donor 2, Donor 3 and Donor 1, respectively) (data not shown). Therefore, mini-pools 1 and 2 were selected for further characterization and all twenty 15-amino acid peptides belonging to these mini-pools were screened using flow cytometry analysis. Among twenty 15-amino acid peptides, IE-1_1–15_MESSAKRKMDPDNPD and IE-1_5–19_AKRKMDPDNPDEGPS induced the highest frequency of IFN-γ producing CD8+ T cells and PBMCs sensitized with these two 15-amino acid peptides showed *in vitro *cytotoxicity against HCMV-infected fibroblast.

Virus-infected human cells can be recognized by CD8+ T cells through antigenic viral protein fragments of 8–12 amino acids in length that are presented on the cell surface in association with HLA class I molecules. Since these smaller peptides can be generated by extracellular processing [25], eleven 9-amino acid peptides with 8 overlapping amino acids and ten 10-amino acid peptides with 9 overlapping amino acids spanning IE-1_1–15 _MESSAKRKMDPDNPD and IE-1_5–19_AKRKMDPDNPDEGPS were synthesized and tested further for identification of HLA-A*2402-restricted HCMV IE-1 epitopes. Among the 21 overlapping peptides, IE-1_3–11_SSAKRKMDP, IE-1_3–12 _SSAKRKMDPD and IE-1_8–16 _KMDPDNPDE induced the greatest frequencies of IFN-γ producing CD8+ T cells. Peptide IE-1_3–12_SSAKRKMDPD induced the highest frequency of IFN-γ producing CD8+ T cells. Although when analyzed by a computer algorithm each of these three peptides scored a low rank estimated half-time of dissociation from the HLA-A24 allele, all three peptides induced high frequencies of polycolonal CD8+ T cells producing IFN-γ; were presented successfully by the HLA-A*2402 allele of HCMV-infected fibroblast cell lines; and induced strong cytotoxicity against HCMV-infected fibroblasts. This suggests that these three peptides are processed naturally and presented successfully *in vitro*.

In conclusion, we have identified a possible HLA-A*2402 CTL epitope, IE-1_3–12 _SSAKRKMDPD, derived from HCMV IE-1 protein using overlapping peptides 15-amino acids in length. This peptide was processed naturally in HCMV-infected human fibroblast and presented successfully on the HLA-A*2402 allele and was well recognized by HCMV-specific polyclonal CD8+ cytotoxic T cells.

## Conclusion

HCMV IE-1_3–12_SSAKRKMDPD is a possible HCMV-specific epitope for vaccination, adoptive immunotherapy, and the monitoring of cellular immune response against HCMV disease in transplant recipients.

## Conflict of interests

The authors declare that they have no competing interests.

## Authors' contributions

JBL designed the research, preformed research, analyzed data, and wrote the paper. HOK designed the research, was responsible for the collection of PBMCs and histocompatibility testing, analyzed data, and wrote the paper. SHJ designed the research, performed research, analyzed data and wrote the paper. JEH performed research, analyzed data, and wrote the paper. SJ performed research, analyzed data and wrote the paper. SGL performed research, analyzed data and wrote the paper. KL designed the research and editing the paper. DFS designed the research and wrote the paper.
